# A Framework for Bus Trajectory Extraction and Missing Data Recovery for Data Sampled from the Internet

**DOI:** 10.3390/s17020342

**Published:** 2017-02-10

**Authors:** Changfei Tong, Huiling Chen, Qi Xuan, Xuhua Yang

**Affiliations:** 1 College of Physics & Electronic Information Engineering, Wenzhou University, Wenzhou 325035, China; chenhuiling_jsj@wzu.edu.cn; 2 College of Information Engineering, Zhejiang University of Technology, Hangzhou 310023, China; xuanqi@zjut.edu.cn; 3 College of Computer Science and Technology, Zhejiang University of Technology, Hangzhou 310023, China; xhyang@zjut.edu.cn

**Keywords:** trajectory processing, anomaly detection, missing data recovery, clustering

## Abstract

This paper presents a novel framework for trajectories’ extraction and missing data recovery for bus traveling data sampled from the Internet. The trajectory extraction procedure is composed of three main parts: trajectory clustering, trajectory cleaning and trajectory connecting. In the clustering procedure, we focus on feature construction and parameter selection for the fuzzy C-means clustering method. Following the clustering procedure, the trajectory cleaning algorithm is implemented based on a new introduced fuzzy connecting matrix, which evaluates the possibility of data belonging to the same trajectory and helps detect the anomalies in a ranked context-related order. Finally, the trajectory connecting algorithm is proposed to solve the issue that occurs in some cases when a route trajectory is incorrectly partitioned into several clusters. In the missing data recovery procedure, we developed the contextual linear interpolation for the cases of missing data occurring inside the trajectory and the median value interpolation for the cases of missing data outside the trajectory. Extensive experiments are conducted to demonstrate that the proposed framework offers a powerful ability to extract and recovery bus trajectories sampled from the Internet.

## 1. Introduction

Urban transportation is being increasingly studied due to growing congestion and its significant impact on people’s daily activities. Currently, the sampling and mining of large-scale traveling data from the real world in order to explore the dynamic properties of traffic systems is becoming a popular research topic. For instance, Zheng et al. [1,2,3,4] sampled trajectories generated by more than 33,000 taxis over a six-month period in Beijing, which contributed to the theory and application of sparse GPS data sensing. By analyzing traffic conditions and driver behaviors, a recommendation system was designed for both taxi drivers and passengers to improve drivers’ pick-up efficiency and reduce passengers’ average wait time. Pan et al. [5,6,7,8,9] investigated human mobility patterns and city dynamics in an urban taxi transportation system by mining the GPS of taxis in Hangzhou, China. Moreover, Liu et al. [10] inferred road maps from the GPS of Shanghai taxis with low resolution and sampling frequency.

In fact, taxis are not the only type of floating probe that can be used to sense urban traffic; rather, the GPS data of urban buses can also be used to detect urban traffic statuses and passengers’ mobility in a city. It is important to note that buses provide a good coverage of cities and run in a fixed road net with better spatial-temporal regularity than do taxis. Bejan et al. [11,12] proposed a statistical model based on the analysis of the sparse GPS data of over 100 buses in the city of Cambridge, and they further evaluated the velocity fields and congestions of the urban traffic. Amaral et al. [13] introduced a tool, called *BusesInRio*to collect and manage bus GPS data in the City of Rio de Janeiro. However, studies on urban bus trajectory processing are reported much less than those on taxi trajectories, especially for large-scale data that take into consideration hundreds or even thousands of buses. The theories and methods developed in the area of data mining for taxi trajectories may not be suitable for urban bus GPS data because buses exhibit more regular trajectories in the road network and are dispatched in a repeated pattern with a repeated cycle. However, buses are often associated with uncertain boarding and alighting times. Due to the severe traffic congestion and the relatively large spatial-temporal deviation of passenger mobility behavior in many cities in China, most current research on the dynamic models of urban bus trajectories is not able to predict bus arrival time with high precision, nor can they optimize a dispatching schedule in a good manner.

In recent years, the Chinese government has upgraded the infrastructure of the urban bus systems in many cities. Buses equipped with GPS devices report real-time location information back to a service center. Several cities, such as Suzhou, Wuxi, Qingdao, Hangzhou, Wenzhou, and so on, have built corresponding websites to better serve passengers, i.e., these websites provide real-time bus arrival information so that passengers can make more flexible travel plans. For researchers, these websites also provide a very good opportunity to collect large-scale bus trajectory data and then to further investigate urban traffic dynamics by data mining methods. One challenge is that there may be a number of erroneous and missing data in the collected data as a result of an unstable GPS signal influenced by high buildings in the city. Additionally, the message packet may be lost as a result of poor mobile communication, which increases the difficulty of trajectory extraction.

Our project concerning urban bus traveling data acquisition from the Internet was started in 2011, and it primarily focused on bus data published from the Suzhou traffic management department [14]. Previous work was published in [15] for the early dataset. However, the method presented in [15] ignored the arrival data of the bus identification, which resulted in incorrect extraction when the trajectories had a short headway or were bunched. Furthermore, the work in [15] did not address the missing data recovery issue. In this paper, a new framework for bus trajectory extraction is developed in a generalized form, which is more efficient in erroneous data detection and more robust in extracting a whole route trajectory than the previous method presented in [15]. The work of this paper consists of two parts: bus trajectory extraction with noise removal and missing data recovery for the extracted trajectories.

In our work, noise removal is based on the anomaly detection method. Outliers are patterns in data that do not conform to a well-defined notion of normal behavior. Anomaly detection refers to the outlier detection problem of identifying the data that are inconsistent with the rest of the set. Anomaly detection is an important problem that has been researched in many diverse research areas and application domains [16]. Numerous anomaly detection (AD) approaches have been presented in relevant literature, including a stochastic model-based approach, such as Gaussian mixture [17], artificial neural networks (ANN) [18], distance-based outlier detection [19], decision function-based approaches, such as one-class support vector machine (SVM) classification [20], the tensor-based method [21], and so on. The types of anomalies can be classified into two categories: point anomalies and contextual anomalies. Contextual anomaly detection seeks to find relationships within datasets where variations in external behavioral attributes well describe the anomalous results in the data [22]. Compared to point anomalies, contextual anomalies detection has been further explored for time series data. The bus trajectories are sequential data that reveal spatial-temporal features. In this sense, we prefer to adopt the contextual anomalies detection for bus trajectories noise removal. In contrast to the traditional AD method for bus trajectories denoising and extracting, this work focuses on erroneous data elimination in addition to the extraction of the trajectories in the form of one trajectory for one route. In this paper, we first apply the fuzzy C-means clustering (FCM) method to split the trajectories into small clusters. Then, we propose a pentagonal fuzzy membership function (MF) based on historical traveling data to measure the probability of two arrival data in one trajectory. The point-to-point MF is extended to the fuzzy connecting MF matrix for measuring the connecting probability of a whole trajectory cluster (also called a trajectory fragment in this paper), and then, AD detection is given based on the MF matrix with prioritized noise removal. When the trajectories are cleaned, a trajectory connecting algorithm is given based on our proposed fuzzy connecting matrix, which helps to connect the trajectory fragments into a whole one-route trajectory.

After the one-route trajectories are extracted, another important work of this paper is to recover the missing data of the trajectories. The missing data recovery is realized by path interpolation. To date, linear interpolation is the easiest, most conservative and most common interpolation method, which assumes that movement follows a straight-line path between two known points [23]. However, straight lines are not consistent with many types of path dynamics, especially for bus trajectories that include consecutive congested and non-congested roads. Other interpolation methods, such as kinematic interpolation [23], cubic Bezier curves [24] and Catmull-Rom curves [25], are also applied in missing data recovery for moving objects. Kinematic interpolation [23] requires speed information and dynamic traveling models. However, the GPS data, such as speed, latitude and longitude, are not provided on the official website in our data acquisition system. Moreover, there are hundreds of lines and thousands of road sections in a city, and the traveling dynamics change at different times of day and for different road sections. Furthermore, it is also cumbersome to determine how to model the traveling dynamics. In this point, kinematic interpolation is not appropriate for our system. In a real system, we have applied the cubic Bezier curves and Catmull-Rom curves in order to test the recovering performance for the sampled data. Unfortunately, the mean absolute error (MAE) of those cubic interpolation methods exhibits a worse performance than the straight linear interpolation in most cases for the sampled station-level sparse arrival data. In this work, we extend the linear interpolation method by considering the historical data and contextual arrival information, which achieves a better performance than that of traditional methods.

The main contributions of this article are summarized as follows:

1. A novel framework of urban bus trajectory extraction is developed, which provides a generalized preprocessing of the raw data sampled from the real-time bus arrival information querying website offered by the Suzhou Transportation Bureau. The trajectory extraction framework consists of three sub-procedures: data clustering to aggregate trajectory fragments, data cleaning to eliminate noisy data in the trajectories and data connecting to merge the fragments into complete one-route trajectories. The proposed framework enables researchers from different regions to sample the urban bus traveling data from the Internet and to extract the trajectories with noise removal, thus providing a new method for urban computing via the traffic data of buses.
2. The fuzzy membership function matrix is first introduced to deal with the problems of contextual anomalies detection, which is applied for noisy data removal in the trajectories. Furthermore, the MF matrix is extended to solve the problem of trajectory fragments connecting with erroneous data tolerance. The proposed MF matrix can be evaluated by the given empirical parameters and can be refined by the sampled data, which is easy to implement and is robust in the trajectory cleaning capability.
3. An efficient scheme that takes into consideration historical data, as well as contextual arrival information is presented for the missing data recovery of the traveling data. Numerous experiments demonstrate that the proposed method outperforms other traditional interpolating methods, such as straight linear interpolation and Catmull-Rom curves.

The remainder of this paper is organized as follows. Section 2 describes how to obtain bus arrival data from the Internet. Section 3 provides some definitions to assist with the problem statement. Section 4 contains major contributions, giving the solutions to infer bus trajectory with outlier elimination and missing data recovery. The experimental results and discussion are presented in Section 5. Finally, conclusions and future work are summarized in Section 6.

## 2. The Bus Arrival Data Acquisition Project

**Figure 1:**
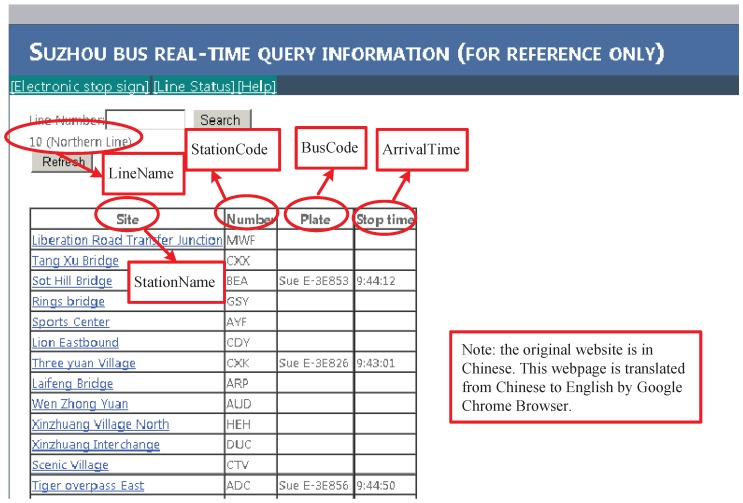
Snapshot of the web page for the bus arrival information on the official website in Suzhou.

We collected bus arrival data in Suzhou from its official website [14]. Figure 1 is a snapshot of the web page (translated from Chinese to English by Chrome Browser), and brief explanations are provided via the red squares and circles. In the system, each bus line is sampled approximately every 30 s and is stored in the Sqlite3 database.

**Figure 2:**
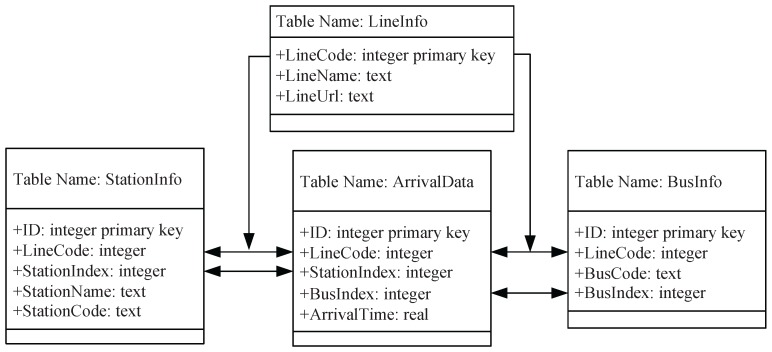
Database design for the urban bus arrival information.

For the original arrival information shown in Figure 1, we designed four types of tables, i.e., “LineInfo”, “StationInfo”, “ArrivalData” and “BusInfo”, to maintain the arrival data in the database as shown in Figure 2. In the table “LineInfo”, when a line is collected, it first queries the table by *LineUrl*. If the querying result returns null, then the *LineName* and *LineUrl* are inserted, and *LineCode* is automatically increased by one for the primary key property. Bus lines with the same line name, but opposite traveling directions are considered to be different lines. Because *LineUrl* is the web page URL of the collected line, it is unique and can be used as the one-to-one mapping of *LineCode*. The remaining three types of tables, “StationInfo”, “ArrivalData” and “BusInfo”, were created and stored in a database for each sampling day. In the database, the field *StationIndex* is a sequenced station index starting at one at the first station along the line route. During the operation days, the public traffic company may change some line route, either temporarily or permanently. Therefore, the stations of a focused line with the same station index may represent different stations on different sampling days, and the table “StationInfo” can help keep track of these changes. The mappings between database fields and web page information are illustrated in Figure 1, where *BusIndex* starts at one for every bus line and is produced by our program according to the appearance order of *BusCode* with respect to the sampling line. *ArrivalTime* is an eight byte float number that is recorded in minutes from the reference at midnight “00:00:00”, e.g., ArrivalTime=9×60+44+12/60=584.2 at arrival time “9:44:12”. The table “ArrivalData” is the main table for arrival data records, which is utilized for investigating bus traffic in our project.

**Figure 3:**
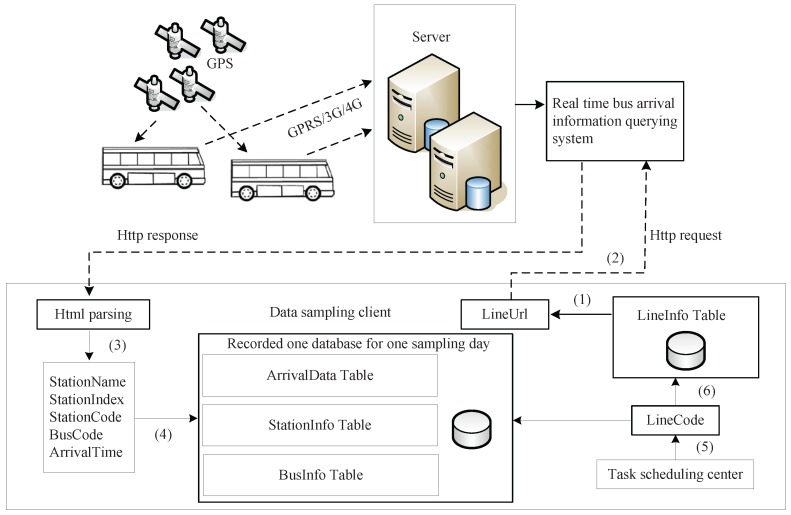
Flowchart of the bus arrival data acquisition project.

The flowchart of our project is illustrated in Figure 3. Almost all of the buses in Suzhou are equipped with GPS devices, and they report their positions to the remote server when they are in service. The server processes the GPS data and finally offers the real-time bus arrival information in the form of a web page, as shown in Figure 1. Users can query the bus arrival data by line name (line number), station name or station code in the website [14]. In the data sampling client, we designed four types of database tables, as shown in Figure 2. Table “LineInfo” was built before the sampling task, which is generated according to the bus lines and the corresponding URLs offered by the website. The task scheduling center schedules which line is to be sampled for the present task. The flowchart is depicted as follows:

1. Query the *LineUrl* by *LineCode* in the “LineInfo” table by the task scheduling center.
2. Query the bus arrival information by http request with the URL bundled to *LineCode*.
3. Parse the HTML from the remote server and extract the arrival data *StationName*, *StationIndex* (processed by program), *StationCode*, *BusCode* and *ArrivalTime* (parsed from text to float number by program) from the web page, as shown in Figure 1.
4. Write the arrival data to the corresponding three tables.
5. Sleep until the next sampling time occurs, and then, update the *LineCode* to the next line by the task scheduling center.
6. Go back to Step 1 and perform the sampling until the task finishing time hits.

In the database, “ArrivalData” is the main table, which is utilized for bus trajectories extraction and traveling time inspection. Assume the *LineCode* for the line shown in Figure 1 is 158 (generated in the database). The web page shown in Figure 1 consists of three arrival events; thus, three records are added to “ArrivalData”, as illustrated in Table 1. In the table, all data are numerical, which is more convenient than text for data mining and scientific calculation.

## 3. Problem Statements

**Definition** **1.** *The bus arrival information matrix extracted from the table “ArrivalData” for line l in one day is denoted by $G^{\left(l\right)}=\{G_{j}^{\left(l\right)}=\left[T_{j},I_{j},B_{j}\right]\left|j=1,\cdots ,\right|G^{\left(l\right)}\left|\right\}$, where l is the line code, $|G^{\left(l\right)}|$ is the size of $G^{\left(l\right)}$ and $G_{j}^{\left(l\right)}$ is the j-th record of the arrival information that consists of ArrivalTime $T_{j}$, StationIndex $I_{j}$ and BusIndex$B_{j}$. For example, the bus arrival information matrix in Table 1 is:*

$$G^{\left(l\right)}=\left[\begin{array}{ccc} 584.2000 & 3 & 1\\ 583.0167 & 7 & 2\\ 584.8333 & 12 & 3 \end{array}\right],\;\mathrm{for}\;l=158$$

**Definition** **2.** The bus arrival information matrix generated by bus m for line l is denoted by $G^{\left(m,l\right)}=\left\{G_{j}^{\left(m,l\right)}=\left[T_{j},I_{j}\right]|\left[T_{j},I_{j},B_{j}\right]\in G^{\left(l\right)},\;B_{j}=m\right\}$. $G^{\left(m,l\right)}$ is a two-dimensional matrix extracted from $G^{\left(l\right)}$. If there are M buses in $G^{\left(l\right)}$, then $G^{\left(l\right)}$ can be reconstructed by $\cup_{m=1}^{M}G^{\left(m,l\right)}$.

**Definition** **3.** The station-level arrival information data generated by bus m for line l at station k are denoted by $G^{\left(k,m,l\right)}=\left\{G_{j}^{\left(k,m,l\right)}=T_{j}|\left[T_{j},I_{j}\right]\in G^{\left(m,l\right)},\;I_{j}=k\right\}$. $G^{\left(k,m,l\right)}$ is a one-dimensional vector extracted from $G^{\left(m,l\right)}$. If there are N stations in the line, then $G^{\left(m,l\right)}$ can be reconstructed by $\cup_{k=1}^{N}G^{\left(k,m,l\right)}$.

**Definition** **4.** The consumed time of a bus from station i to station j in the driving direction for line l is denoted by $t_{ij}^{\left(l\right)}$. $t_{ij}^{\left(l\right)}$ includes the traveling time on the corresponding road, the waiting time caused by traffic signals at a crossroad and the dwell time at stations i to j−1 caused by passengers’ boarding and alighting behaviors. $t_{ij}^{\left(l\right)}$ is an important feature for urban buses’ traveling behaviors, because it provides a scratch view of the crowdedness for the corresponding road. $t_{ij}^{\left(l\right)}$ can be utilized to investigate the urban traffic, the spatial-temporal property of the urban bus, the arrival time prediction, etc. Unfortunately, $t_{ij}^{\left(l\right)}$ cannot be directly derived from either $G^{\left(l\right)}$ or $G^{\left(m,l\right)}$ because the records in $G^{\left(l\right)}$ lack the route information. Thus, before calculating $t_{ij}^{\left(l\right)}$, it is necessary to extract the one-route trajectories in $G^{\left(l\right)}$.

**Definition** **5.** The one-route trajectories set generated by bus m for line l is denoted by $S^{\left(m,l\right)}$, where one-route trajectory represents the trajectory generated by a bus from the first station to the terminal station in one round. The k-th one-route trajectory in $S^{\left(m,l\right)}$ is denoted by $S^{\left(k,m,l\right)}\in S^{\left(m,l\right)}$, and the one-route trajectories set generated from all M buses in line l is denoted by $S^{\left(l\right)}=\cup_{m=1}^{M}S^{\left(m,l\right)}$. Once $S^{\left(l\right)}$ is derived from $G^{\left(l\right)}$, it is easy to obtain $t_{ij}^{\left(l\right)}$ by $T_{j1}-T_{j2}$ with $I_{j1}=j$ and $I_{j2}=i$ in $S^{\left(k,m,l\right)}\in S^{\left(l\right)}$, where $I_{j1}$ and $I_{j2}$ are the station indices.

**Figure 4:**
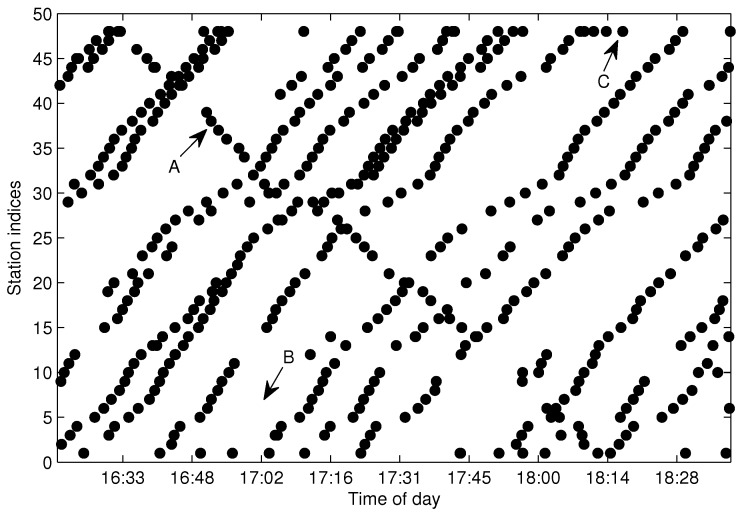
Bus arrival events collected in $G^{\left(l\right)}$ for l=130 in Suzhou. Sampling date: 2 August 2012.

The process of generating $S^{\left(l\right)}$ from $G^{\left(l\right)}$ is referred to as trajectories extraction throughout this paper. As pointed out in [1], spatial trajectories are never perfectly accurate due to sensor noise and other factors. In reality, there will be an amount of missing data and abnormal data in $G^{\left(l\right)}$ caused by erroneous GPS signal, communication failure and data sampling fault, which causes trajectories extraction to be a difficult task. Figure 4 shows the bus arrival events collected in $G^{\left(l\right)}$ for l=130 (Line 10 southward) from the Suzhou transportation website [14] with a sampling period of 30 s. Case A in the figure shows an abnormal trajectory, which was caused by the bus traveling in the opposite direction. A reasonable explanation for Case A is that the bus driver forgot to switch the GPS reporting device to the right direction when the bus changed route direction. The wrong direction trajectories need to be distinguished and eliminated from $G^{\left(l\right)}$ in the trajectory extraction process. Case B shows a severe data missing problem for the designated route. The incomplete data will impede the inference of $S^{\left(l\right)}$. Case C shows the GPS over-reporting at the terminal station. Case C is caused by the fact that the GPS device continues to report more than one GPS position to the service after the bus reaches its destination. The situation at the first station or some other stations that have a long dwell time may also result in more than one position being reported to the service, which will cause the trajectory extraction to be more complicated. When over-reporting and wrong direction reporting occur in adjacent time slots, it is difficult to extract the trajectories by the direct search method.

**Figure 5:**
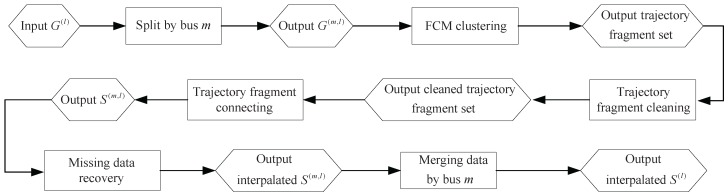
Framework of the data processing for trajectories extraction and missing data recovery.

In this paper, we focus on two problems associated with arrival data processing, where the data were collected from the Internet. The first concerns trajectories extracted from $G^{\left(l\right)}$ with outlier elimination, and the second one concerns missing data recovery for the extracted trajectories. The framework of the data processing is shown in Figure 5. Firstly, the input arrival data $G^{\left(l\right)}$ are split by different buses *m* to produce $G^{\left(m,l\right)}$. Secondly, a fuzzy C-means clustering (FCM) method is applied to cluster the data in $G^{\left(m,l\right)}$, and the trajectory fragment set is generated. Then, the outliers in the trajectory fragments are eliminated by the proposed cleaning algorithm. Next, a trajectory fragment connecting method is applied to connect the cleaned trajectory fragments into one-route trajectories set $S^{\left(m,l\right)}$. Finally, the missing data in $S^{\left(m,l\right)}$ are recovered, and the complete one-route trajectories set $S^{\left(l\right)}$ is merged by the interpolated $S^{\left(m,l\right)}$.

## 4. Trajectories Extraction and Missing Data Recovery

### 4.1. Fuzzy C-Means Clustering

Clustering and anomaly detection are extensively studied for trajectory mining applications [1,26]. Furthermore, the two techniques are often used in conjunction [27]. In our project, we first cluster the arrival data into a trajectory fragment if those data should be grouped into the same route for a bus. Then, the anomaly detection method is applied to check whether there exist outliers in the trajectory fragment and removes the erroneous data according to the contextual relationship of the data. We adopt fuzzy C-means clustering (FCM) as the trajectory clustering method. FCM is a soft clustering method and was proposed by Bezdek [28]. In the FCM method, the data $X=\{X_{1},X_{2},\cdots X_{n}\}$ are partitioned into *c* fuzzy sets by minimizing the objective function as follows:

(1) $$J_{a}\left(U,V\right)=\sum_{i=1}^{n}\sum_{j=1}^{c}{\left(u_{ij}\right)}^{a}\left|\right|X_{i}-V_{j}{\left|\right|}^{2}$$

where a>1 is the fuzzification coefficient, *V* is the prototype matrix with $V_{j}$ denoting the *j*-th cluster prototype, *U* is the partition matrix with $u_{ij}$ denoting the membership value for $X_{i}$ classified to cluster *j* and $\left|\right|X_{i}-V_{j}\left|\right|$ is a distance function. Usually, the Euclidean distance is considered for $\left|\right|X_{i}-V_{j}\left|\right|$. The membership $u_{ij}$ is constrained by:

(2) $$\left\{\begin{array}{c} 0\leq u_{ij}\leq 1,\;\forall i,j\\ 0<\sum_{i=1}^{n}u_{ij}\leq n,\;\forall i\\ \sum_{j=1}^{c}u_{ij}=1,\;\forall j \end{array}\right.$$

The minimization of $J_{a}\left(U,V\right)$ with Constraint (2) gives [28]:

(3) $$V_{j}=\frac{\sum_{i=1}^{n}u_{ij}^{a}X_{i}}{\sum_{i=1}^{n}u_{ij}^{a}}$$

(4) $$u_{ik}=\frac{1}{\sum_{j=1}^{c}{\left(\frac{\left|\right|X_{i}-V_{k}\left|\right|}{\left|\right|X_{i}-V_{j}\left|\right|}\right)}^{\frac{2}{a-1}}}$$

In practice, the partition matrix *U* and prototype matrix *V* are updated in an iterative way by Formulas (3) and (4) until the change of objective function $\Delta J_{a}\left(U,V\right)$ between two consecutive iteration is less than a predefined tolerance or the maximum iterating times reached.

In this paper, we utilize the FCM method to cluster the arrival data with the objective that the data in the same cluster should belong to the same route. Of course, the best result is one cluster for one-route trajectory. However, due to the disturbance of outliers and data missing problems, the actual clusters are not always equal to the route trajectories.

### 4.2. Feature Construction for FCM

Feature construction is an important work in FCM. The arrival data $G^{\left(l\right)}$ contains three-dimensional information generated by line *l*. Usually, one line consists of several buses that can be distinguished by $B_{j}$ in $G^{\left(l\right)}$. In the sense of trajectory clustering, we wish to partition the arrival data generated by one route in $G^{\left(l\right)}$ into one cluster. Hence, the arrival data generated by different buses should be naturally partitioned into different trajectory clusters. However, if we take $G_{j}^{\left(l\right)}\in G^{\left(l\right)}$ as the input feature of *X* for FCM, the distance $\left|\right|G_{i}^{\left(l\right)}-G_{j}^{\left(l\right)}\left|\right|$ for i≠j cannot effectively measure the difference between different trajectories. On the other hand, the two-dimensional information $G^{\left(m,l\right)}$ extracted from $G^{\left(l\right)}$ is better for trajectory clustering, since all arrival data in $G^{\left(m,l\right)}$ are generated by the same bus *m*, and arrival data generated by different buses are naturally separated by $G^{\left(m,l\right)}$ with different values of *m*. In practice, there are more abnormal arrival data at the first and final stations than other stations; we eliminate the FF-data (arrival data of the first and final stations) before trajectories extraction for the sake of reducing the negative effect introduced by abnormal data.

**Figure 6:**
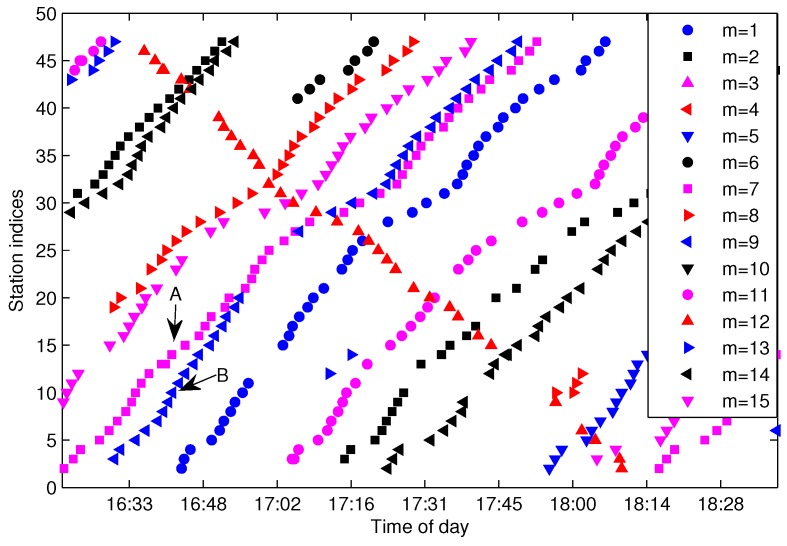
Bus arrival events grouped by $G^{\left(m,l\right)}$ for the same data $G^{\left(l\right)}$ as shown in Figure 4.

Figure 6 shows the arrival events grouped by $G^{\left(m,l\right)}$ after eliminating the FF-data for the same dataset shown in Figure 4. As illustrated in Figure 6, Trajectories A and B can be efficiently distinguished by $G^{\left(m,l\right)}$; however, quite differently, they are difficult to partition from $G^{\left(l\right)}$ without the bus-code information when the trajectories are in a short headway or bunched, as shown in Figure 4. If we adopt the method presented in [15], Trajectories A and B will not be extracted correctly. Hence, grouping arrival data by $G^{\left(m,l\right)}$ instead of $G^{\left(l\right)}$ is a sensible choice for the purpose of trajectory extraction, especially for the trajectories in a short headway. Though $G^{\left(m,l\right)}$ can separate trajectories from different buses, the arrival data in $G^{\left(m,l\right)}$ still consist of several traveling routes and need to be further partitioned from each other.

**Figure 7:**
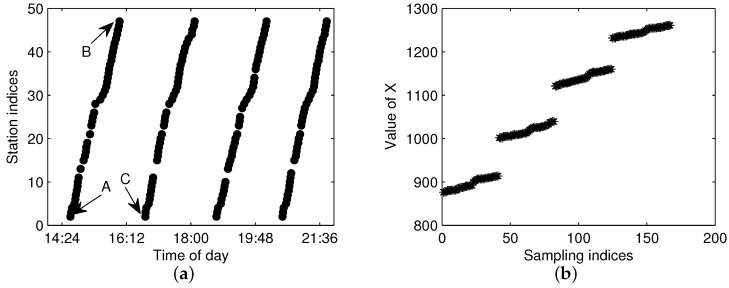
The constructed input feature of $G^{\left(m,l\right)}$ without abnormal data. (**a**) Arrival events; (**b**) constructed *X*.

The FCM clustering of $G^{\left(m,l\right)}$ is aimed to separate the arrival data by one cluster for each one-route trajectory. Figure 7a shows the arrival events by the bus with m=1 for the same line and sampling date as in Figure 4. From the figure, one can see that there are total of four traveling routes for bus m=1, which means that the work of trajectory extraction is to extract the four trajectories and add them into $S^{\left(m,l\right)}$. The first problem for FCM is how to measure the distance of trajectory data in order to get a large distance between different trajectories and a small distance in the same trajectory. Though traditional Euclidean distance is widely used in clustering, it is not a good metric for our FCM clustering. We take three points in Figure 7a as an example, as shown in the figure, Points *A* and *B* are in the same trajectory; however, the Euclidean distance between *A* and *C* (points from different trajectories) is smaller than the distance between *A* and *B*, which will result in the wrong clustering for FCM.

For trajectory clustering by FCM, we construct an enhanced distance metric by considering the traveling time along the route direction. Let the *j*-th record in $G^{\left(m,l\right)}$ be $G_{j}^{\left(m,l\right)}=\left[T_{j},\;I_{j}\right]$; then, the input feature $X_{j}$ for $G_{j}^{\left(m,l\right)}$ is:

(5) $$X_{j}=T_{j}-R\left(I_{j}\right)$$

where $T_{j}$ is the arrival time for record *j*, $I_{j}$ is the arrival station index in line *l* and $R(I_{j})$ is a linear function of the traveling time from the first station to station $I_{j}$. For most stations in the studied city, the uncongested traveling time between two consecutive stations is about 2 min, and $R(I_{j})$ is selected as half of the traveling time; hence, a robust and convenient choice for the initial value of $R(I_{j})$ is:

(6) $$R\left(I_{j}\right)=I_{j}$$

Once the trajectories of a studied line are extracted, $R(I_{j})$ can be refined according to the historical traveling time derived from the trajectories. Figure 7b shows the constructed input feature *X* of $G^{\left(m,l\right)}$. As shown in the figure, the constructed *X* is a one-dimensional datum, which can split the traveling data of $G^{\left(m,l\right)}$ into four clusters well, with each cluster mapped to a one-route trajectory. The illustrated case in Figure 7 is suitable for the well-conditioned traveling data without noise. However, there may exist some abnormal data as depicted in Figure 4; the input feature constructed by Equations (5) and (6) thus may reduce the separable performance when the arrival data in $G^{\left(m,l\right)}$ contain more trajectories in the opposite direction than those in the correct direction. In order to enhance the separability for the case of the wrong direction trajectories, the $R(I_{j})$ in Equation (6) is modified by:

(7) $$R_{b}\left(I_{j}\right)=-R\left(I_{j}\right)$$

and $X_{j}$ in Equation (5) is modified as:

(8) $$X_{j}=T_{j}-R_{b}\left(I_{j}\right)=T_{j}+R\left(I_{j}\right)$$

The *X* constructed by Equation (5) is called the forward clustering feature, and the one constructed by Equation (8) is called the backward clustering feature in this paper. Accordingly, we call the fuzzy C-means clustering with the forward clustering feature as forward FCM (F-FCM) and the one with backward clustering feature as backward FCM (B-FCM).

### 4.3. Parameters Selection for FCM

The most important parameters for our trajectory extraction in the procedure of FCM are the number of fuzzy sets *c* and initial prototypes. In practice, the counts of trajectories always change by different buses in different sampling days and need to be determined automatically. We count the trajectories based on station scanning, which is depicted as follows:

(9) $$c_{0}=\max_{k}\;\;\left|G^{\left(k,m,l\right)}\right|,\;\;k=2,3,\cdots ,N-1$$

where $c_{0}$ is the count of trajectories for $G^{\left(m,l\right)}$, *N* is the number of stations in line *l*, |·| is the size of the set and $G^{\left(k,m,l\right)}$ is defined in Definition 3. Since there are more abnormal arrival events at the first and final stations, the scanning process is started from the second station and terminated before the final station to reduce the effect of outliers. Actually, the scanned $c_{0}$ may be less than the actual count (denoted by $c_{0}^{*}$) in some cases caused by data missing or be greater than $c_{0}^{*}$ in other cases caused by over-reporting. We prefer to set $c>c_{0}^{*}$ to avoid partitioning two different trajectories into one cluster. In our extraction procedure, *c* is set by:

(10) $$c=[\alpha c_{0}],\;\alpha >1$$

where $\alpha \in R^{+}$ is the gain of *c* over $c_{0}$ and is chosen by α∈[1.3,2.0] in most cases. In Equation (10), $\left[\alpha c_{0}\right]$ is an integer operation rounding $\alpha c_{0}$ to the nearest integer less than or equal to $\alpha c_{0}$.

The iterative algorithm for FCM clustering is a local optimization procedure; hence, different initial prototypes may result in different partitions. One property of the constructed feature *X* is that both *X* and trajectories are ordered in arrival time. Inspired by the time-ordered property, we proposed an initial prototype selection method as follows:

(11) $$\left\{\begin{array}{c} V_{i}^{\left(0\right)}=X_{k}\\ k=[\frac{\left|X|\cdot (i-0.5\right)}{c}] \end{array},\;\;i=1,2,\cdots ,c\right.$$

where $V_{i}^{\left(0\right)}$ is the *i*-th prototype in the initial prototype vector $V^{\left(0\right)}$, $X_{k}$ is the *k*-th element in *X* and *c* is the number of clusters. If all clusters are of the same size, the prototypes selection approach proposed in Equation (11) will choose the center of the cluster as the initial prototype, which will reduce the clustering iterations. In this sense, the proposed initial prototypes selection method is better than random selection with the advantage that it costs less iterations in most cases when the sizes of the sampled trajectories are nearly equal.

### 4.4. The FCM Clustering Algorithm

The F-FCM clustering algorithm is shown in Algorithm 1, which is an iterative algorithm. By default, we set α=1.8, $K_{\max}=100$, δ=0.01 in the algorithm. The B-FCM clustering algorithm is similar to F-FCM, except for the feature generating in Step (a3).

Let $G_{c}^{\left(m,l\right)}=\cup_{j=1}^{c}G_{c}^{\left(j,m,l\right)}$ be the output of the aggregated cluster set by FCM, where $G_{c}^{\left(j,m,l\right)}$ is the *j*-th cluster in $G_{c}^{\left(m,l\right)}$. The final FCM clustering algorithm is an optimal partition selection between F-FCM and B-FCM according to the objective function, which is given by Algorithm 2. In Algorithm 2, $G_{c}^{\left(m,l\right)}$ is generated according to the partition matrix *U*.

**Algorithm 1** F-FCM clustering algorithm.**Input:**
$G^{\left(m,l\right)}$;**Output:**
$U^{F-FCM}$ (the partition matrix), $V^{F-FCM}$ (the prototypes set), $J_{a}^{F-FCM}$ (the objective function);**Process:**
(a1) get $c_{0}$ by Equation (9);(a2) set *α* and get *c* by Equation (10);(a3) generate the clustering feature *X* by Equation (5);(a4) initiate the prototypes set $V^{\left(0\right)}$ by Equation (11), get $U^{\left(0\right)}$ by Equation (4), get $J_{a}^{\left(0\right)}≜J_{a}\left(U^{\left(0\right)},V^{\left(0\right)}\right)$ with Equation (1);(a5) set the maximum FCM iteration $K_{\max}$, error tolerance *δ* and execute the following iterative loop:    **for** *i*← 1 **to** $K_{\max}$ **do**        get $V^{\left(i\right)}$ by $U^{\left(i-1\right)}$ with Equation (3);        get $U^{\left(i\right)}$ by $V^{\left(i\right)}$ with Equation (4);        get $J_{a}^{\left(i\right)}$ by Equation (1);        **if** $|J_{a}^{\left(i\right)}-J_{a}^{\left(i-1\right)}|<\delta$ **then**           break;        **end if**    **end for**(a6) set $U^{F-FCM}=U^{\left(i\right)}$, $V^{F-FCM}=V^{\left(i\right)}$, $J_{a}^{F-FCM}=J_{a}^{\left(i\right)}$ for the last iteration *i* in Step (a5).

**Algorithm 2** FCM clustering algorithm.**Input:**
$G^{\left(m,l\right)}$, *N* (the number of data in $G^{\left(m,l\right)}$, i.e., $N=|G^{\left(m,l\right)}|$);**Output:**
*U* (the partition matrix), $G_{c}^{\left(m,l\right)}$ (the aggregated clusters);**Process:**
(a1) get $U^{F-FCM}$, $V^{F-FCM}$, $J_{a}^{F-FCM}$ by F-FCM clustering;(a2) get $U^{B-FCM}$, $V^{B-FCM}$, $J_{a}^{B-FCM}$ by B-FCM clustering;(a3) clustering method determination:    **if** $J_{a}^{F-FCM}\leq J_{a}^{B-FCM}$ **then**        set $U=U^{F-FCM}$;    **else**        set $U=U^{B-FCM}$;    **end if**(a4) partition loop:    **for** *i*← 1 **to** *N* **do**        **if** $u_{ij}=\max_{k=1,2,\cdots ,c}\;u_{ik}$ **then**           partition $G_{i}^{\left(m,l\right)}$ (the *i*-th data in $G^{\left(m,l\right)}$) into $G_{c}^{\left(j,m,l\right)}$;        **end if**    **end for**(a5) generate $G_{c}^{\left(m,l\right)}$ by $G_{c}^{\left(m,l\right)}=\cup_{j=1}^{c}G_{c}^{\left(j,m,l\right)}$.

### 4.5. Trajectory Fragment Cleaning

The aggregated $G_{c}^{\left(m,l\right)}$ from $G^{\left(m,l\right)}$ by FCM is called the trajectory fragments set in this paper. When the trajectory fragments set $G_{c}^{\left(m,l\right)}$ is generated by Algorithm 2, the following procedure is about outlier elimination, which is also called trajectory fragment cleaning in this paper.

**Figure 8:**
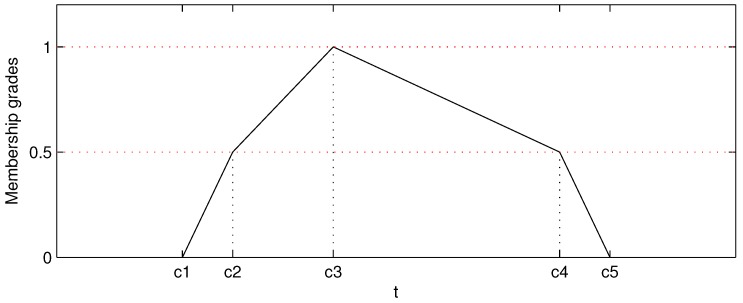
The pentagonal fuzzy membership function.

We utilize fuzzy membership function (MF) to describe the relationship of arrival data in a trajectory, i.e., the introduced MF in our AD procedure describes the connecting possibility between two arrival events $G_{i}^{\left(m,l\right)}$ and $G_{k}^{\left(m,l\right)}$ to be classified into the same trajectory. In fuzzy set theory, most common membership functions are triangular, trapezoidal, Gaussian and bell-shaped [29,30]. In a more generalized viewpoint, the triangular and trapezoidal functions are in the forms of polygons, which are described by three parameters for the triangular and four parameters for the trapezoidal. In this paper, we extend the triangular to pentagonal MF, which is depicted as follows:

(12) $$u\left(t\right)=\left\{\begin{array}{cc} 0\;, & t<c_{1}\\ \frac{t-c_{1}}{2(c_{2}-c_{1})}\;, & c_{1}\leq t<c_{2}\\ \frac{1}{2}+\frac{t-c_{2}}{2(c_{3}-c_{2})}\;, & c_{2}\leq t<c_{3}\\ \frac{1}{2}+\frac{c_{4}-t}{2(c_{4}-c_{3})}\;, & c_{3}\leq t<c_{4}\\ \frac{c_{5}-t}{2(c_{5}-c_{4})}\;, & c_{4}\leq t\leq c_{5}\\ 0\;, & t>c_{5} \end{array}\right.$$

where u(t) is the fuzzy MF of two arrival events to be connected into one trajectory and $c_{1},c_{2},\cdots ,c_{5}$ are the five parameters describing the shape of pentagonal MF as shown in Figure 8. For connecting possibility evaluation, the *t* in Equation (12) is the directional station-unit traveling time between $G_{i}^{\left(m,l\right)}$ and $G_{k}^{\left(m,l\right)}$, which is calculated as follows:

(13) $$t=\left\{\begin{array}{cc} \frac{T_{i}-T_{k}}{I_{i}-I_{k}}\;, & I_{i}\neq I_{k}\\ 0\;, & I_{i}=I_{k}\; \end{array}\right.$$

where $G_{i}^{\left(m,l\right)}=\left[T_{i},I_{i}\right]$ and $G_{k}^{\left(m,l\right)}=\left[T_{k},I_{k}\right]$.

If the trajectories for the studied lines have already been extracted, we can utilize the historical traveling time *t* to determinate the parameters of the pentagonal MF by:

(14) $$\left\{\begin{array}{c} c_{1}=\eta_{\min}c_{2}\\ c_{2}=\min\left(t\right)\\ c_{3}=\mathrm{median}\left(t\right)\\ c_{4}=\max\left(t\right)\\ c_{5}=\eta_{\max}c_{4} \end{array}\right.$$

where $\eta_{\min}<1$ is the lower bound coefficient and $\eta_{\max}>1$ is the upper bound coefficient, which control the extended range of *t*. Compared with the triangular MF, the pentagonal MF has the benefits of more flexible slope control. For data-based knowledge, u(t) should be in high connecting possibility (u(t)≥0.5) for all *t* in the historical data, and u(t)=1 (the highest connecting possibility) when *t* is the median of the historical data. Moreover, the historical data cannot reveal all cases in the future, so the data range should be extended from historical data to make a better coverage for the future. Furthermore, the extensions are not symmetric for the lower bound and upper bound. The shortest traveling time is restricted by the highest vehicle velocity and distance between two stations, which can be expected once the shortest distance between two adjacent stations is given. Hence, there is a slight difference between min(t) derived by historical data and the actual one in the future. However, the longest traveling time will be affected by traffic jam, traffic accident and other factors, which should be evaluated in a more conservative way. Actually, the *t* in Equation (13) is dependent on bus lines, station indices, date types and time of day. For the trajectory extraction work, it is hard to consider all of the factors when extracting hundreds of lines with millions of arrival events generated in one day. We prefer to evaluate u(t) for all arrival events with a set of empirical parameters, which is given as follows:

(15) $$C_{M}=\left[\begin{array}{ccccc} 0.20 & 0.50 & 2.00 & 16.00 & 25.00\\ 0.40 & 0.80 & 2.00 & 12.00 & 17.00\\ 0.40 & 0.80 & 2.00 & 10.00 & 14.00\\ 0.50 & 0.85 & 2.00 & 8.00 & 11.50\\ 0.50 & 0.85 & 2.00 & 7.00 & 9.40\\ 0.55 & 0.90 & 2.00 & 6.00 & 8.20\\ 0.55 & 0.90 & 2.00 & 5.50 & 7.20 \end{array}\right]$$

where the *n*-th row of $C_{M}$ refers to the parametrical set of u(t) with $n=|I_{i}-I_{k}|$ in Equation (13) and the *j*-th column of $C_{M}$ is the parameter $c_{j}$ in Equation (12). When $|I_{i}-I_{k}|$ exceeds the maximum row index of $C_{M}$, the parameters for evaluating u(t) are selected by the last row of $C_{M}$. By using $C_{M}$ given in Equation (15), the u(t) between any two arrival events in a trajectory can be evaluated.

Let $U^{c}\in R^{N\times N}$ be the fuzzy connecting MF matrix evaluated for a trajectory fragment $G_{c}^{\left(j,m,l\right)}$ with *N* arrival events, where $U_{ik}^{c}$ is the u(t) between the *i*-th and *k*-th arrival data when i≠k or $U_{ik}^{c}=1$ when i=k.

Let ${\hat{G}}_{c}^{\left(j,m,l\right)}\subseteq G_{c}^{\left(j,m,l\right)}$ be the cleaned trajectory fragment from $G_{c}^{\left(j,m,l\right)}$ and ${\hat{U}}^{c}$ the cleaned fuzzy connecting MF matrix generated from ${\hat{G}}_{c}^{\left(j,m,l\right)}$. We take the trajectory fragment cleaning as an optimization problem as follows:

(16) $$\max|{\hat{G}}_{c}^{\left(j,m,l\right)}|\;s.t.\;\left\{\begin{array}{c} {\hat{U}}_{ik}^{c}>u_{\min},\;\forall {\hat{U}}_{ik}^{c}\in {\hat{U}}^{c}\\ \mathrm{avg}({\hat{U}}^{c})\;\mathrm{is}\;\mathrm{maximized} \end{array}\right.$$

where $|{\hat{G}}_{c}^{\left(j,m,l\right)}|$ is the size of ${\hat{G}}_{c}^{\left(j,m,l\right)}$, $\mathrm{avg}({\hat{U}}^{c})$ is the average of the elements in ${\hat{U}}^{c}$ and $u_{\min}$ is the connecting possibility threshold, which is given by empirical knowledge. We set $u_{\min}=0.3$ for all arrival data in the project. For a cleaned trajectory fragment ${\hat{G}}_{c}^{\left(j,m,l\right)}$, every arrival datum in ${\hat{G}}_{c}^{\left(j,m,l\right)}$ should be connected, so all of the elements in ${\hat{U}}^{c}$ should be greater than the predefined threshold $u_{\min}$. On the other hand, any data in ${\hat{G}}_{c}^{\left(j,m,l\right)}$ should not be misclassified as noisy data, which is guaranteed by the maximization of $|{\hat{G}}_{c}^{\left(j,m,l\right)}|$.

Let ${\bar{N}}_{i}^{c}$ be the anomalous indexing of the *i*-th data in $G_{c}^{\left(j,m,l\right)}$, which is the count of $u_{ik}$ satisfying $u_{ik}\leq u_{\min}$ for k=1,2,⋯N, and ${\bar{N}}^{c}$ the anomalous indexing vector generated by ${\bar{N}}^{c}={\left[{\bar{N}}_{1}^{c},{\bar{N}}_{2}^{c},\cdots {\bar{N}}_{N}^{c}\right]}^{T}$.

Let ${\hat{G}}_{c}^{\left(m,l\right)}=\cup_{j=1}^{c}{\hat{G}}_{c}^{\left(j,m,l\right)}$ be the set of the cleaned trajectory fragments.

Let ${\hat{R}}_{c}^{\left(j,m,l\right)}$ be the noisy dataset generated from $G_{c}^{\left(j,m,l\right)}$ by the trajectory fragment cleaning algorithm, and ${\hat{R}}_{c}^{\left(m,l\right)}=\cup_{j=1}^{c}{\hat{R}}_{c}^{\left(j,m,l\right)}$. By the definition, one gets $G_{c}^{\left(j,m,l\right)}={\hat{G}}_{c}^{\left(j,m,l\right)}\cup {\hat{R}}_{c}^{\left(j,m,l\right)}$, and $G_{c}^{\left(m,l\right)}={\hat{G}}_{c}^{\left(m,l\right)}\cup {\hat{R}}_{c}^{\left(m,l\right)}$.

The solution of Problem (16) is started by checking the noisy data with maximum anomalous indexing and deleting it from $G_{c}^{\left(j,m,l\right)}$ recursively. The detailed solution of Problem (16) is given by Algorithm 3.

**Algorithm 3** Trajectory fragment cleaning algorithm.**Input:**
$G_{c}^{\left(j,m,l\right)}$, $C_{M}$, $u_{\min}$;**Output:**
${\hat{G}}_{c}^{\left(j,m,l\right)}$, ${\hat{U}}^{c}$, ${\hat{R}}_{c}^{\left(j,m,l\right)}$;**Process:**
(a1) set a temporary noisy set ${\hat{R}}_{c}=⌀$;(a2) get $U^{c}$ of $G_{c}^{\left(j,m,l\right)}$ with $C_{M}$;(a3) get ${\bar{N}}^{c}$ from $U^{c}$ with $u_{\min}$;    **if** ${\bar{N}}^{c}=0$ **then**        go to (a5);    **else**        go to (a4);    **end if**(a4) find the index *k* in ${\bar{N}}^{c}$ with the maximum anomalous indexing;    **if** *k* is not unique **then**        choose $k^{*}$ be the one in *k* with the minimum row summation;    **else**        set $k^{*}=k$;    **end if**    update ${\hat{R}}_{c}$ by adding the $k^{*}$-th data in $G_{c}^{\left(j,m,l\right)}$ into ${\hat{R}}_{c}$;    delete the $k^{*}$-th data in $G_{c}^{\left(j,m,l\right)}$, delete the $k^{*}$-th row and $k^{*}$-th column in $U^{c}$;    go to (a3);(a5) output the cleaned trajectories:    **if** $|G_{c}^{\left(j,m,l\right)}|>1$ **then**        set ${\hat{G}}_{c}^{\left(j,m,l\right)}=G_{c}^{\left(j,m,l\right)}$, and ${\hat{U}}^{c}=U^{c}$;    **else**        set ${\hat{G}}_{c}^{\left(j,m,l\right)}=⌀$, and ${\hat{U}}^{c}=⌀$;    **end if**    set ${\hat{R}}_{c}^{\left(j,m,l\right)}={\hat{R}}_{c}$.

### 4.6. Trajectory Fragments Connecting

In some cases, the one-route trajectory may be broken into two or more trajectory fragments. Hence, when generating $S^{\left(m,l\right)}$ from ${\hat{G}}_{c}^{\left(m,l\right)}$, some trajectories need to be connected to form a one-route trajectory. The process of generating $S^{\left(m,l\right)}$ from ${\hat{G}}_{c}^{\left(m,l\right)}$ is named trajectory fragments connecting (TFC) in this paper. We introduce the fuzzy connecting matrix $U^{F}$ for the processing of TFC. We denote $u_{ij}^{F}$ as the *i*-th row and *j*-th column element in $U^{F}$, which is defined as follows:

(17) $$u_{ij}^{F}=\left\{\begin{array}{cc} \mathrm{avg}({\hat{U}}^{\left(i\cup j\right)}) & \mathrm{if}\;G^{\left(i\cup j\right)}\;\mathrm{satisfying}\;\mathrm{Rule}\;1\\ 0 & \mathrm{others} \end{array}\right.$$

where $G^{\left(i\cup j\right)}={\hat{G}}_{c}^{\left(i,m,l\right)}\cup {\hat{G}}_{c}^{\left(j,m,l\right)}$, ${\hat{U}}^{\left(i\cup j\right)}$ is the fuzzy connecting MF matrix generated from ${\hat{G}}^{\left(i\cup j\right)}$, ${\hat{G}}^{\left(i\cup j\right)}$ is the cleaned trajectory from $G^{\left(i\cup j\right)}$ and $\mathrm{avg}({\hat{U}}^{\left(i\cup j\right)})$ is the average value of the elements in ${\hat{U}}^{\left(i\cup j\right)}$. ${\hat{U}}^{\left(i\cup j\right)}$ and ${\hat{G}}^{\left(i\cup j\right)}$ are generated by Algorithm 3 for the input $G^{\left(i\cup j\right)}$. Rule 1 is described as follows:

(18) $$\left\{\begin{array}{c} |{\hat{R}}^{\left(i\cup j\right)}|<\min\{n_{\tau },|{\hat{G}}_{c}^{\left(i,m,l\right)}\left|,\right|{\hat{G}}_{c}^{\left(j,m,l\right)}\left|\right\}\;\\ \min\left({\hat{U}}^{\left(i\cup j\right)}\right)>u_{\min}\;\\ {\hat{G}}_{c}^{\left(i,m,l\right)}\;\mathrm{is}\;\mathrm{ahead}\;\mathrm{of}\;{\hat{G}}_{c}^{\left(j,m,l\right)}\;\mathrm{in}\;{\hat{G}}^{\left(i\cup j\right)} \end{array}\right.$$

where $|{\hat{R}}^{\left(i\cup j\right)}|$ is the size of noisy dataset ${\hat{R}}^{\left(i\cup j\right)}$ generated from $G^{\left(i\cup j\right)}$ by Algorithm 3, $n_{\tau }$ is the predefined parameter to control the maximum number of noisy data when considering ${\hat{G}}_{c}^{\left(i,m,l\right)}$ and ${\hat{G}}_{c}^{\left(j,m,l\right)}$ might be connected and $u_{\min}$ is as defined in Problem (16). We set $n_{\tau }=3$ in most cases for the TFC processing. In Constraint (18), ${\hat{G}}_{c}^{\left(i,m,l\right)}$ is ahead of ${\hat{G}}_{c}^{\left(j,m,l\right)}$ in ${\hat{G}}^{\left(i\cup j\right)}$, which means that all of the arrival events belonging to ${\hat{G}}_{c}^{\left(i,m,l\right)}$ in ${\hat{G}}^{\left(i\cup j\right)}$ happen before the other events in ${\hat{G}}^{\left(i\cup j\right)}$. From Equation (17) and Constraint (18), one can see that $U^{F}$ is not a symmetric matrix; moreover, if $u_{ij}^{F}>0$, we will definitely have $u_{ji}^{F}=0$.

In the processing of TFC, it is better to select the pair of fragments in ${\hat{G}}_{c}^{\left(m,l\right)}$ with the maximum connecting possibility for connection, which is easily conducted by finding $u_{\max}^{F}$ (the maximum element in $U^{F}$). Once ${\hat{G}}_{c}^{\left(i,m,l\right)}$ and ${\hat{G}}_{c}^{\left(j,m,l\right)}$ can be connected, we set ${\hat{G}}_{c}^{\left(i,m,l\right)}={\hat{G}}^{\left(i\cup j\right)}$ and delete ${\hat{G}}_{c}^{\left(j,m,l\right)}$ in ${\hat{G}}_{c}^{\left(m,l\right)}$. In this way, $U^{F}$ is partly updated in each connection round. The detail of TFC is given by Algorithm 4.

**Algorithm 4** Trajectory fragments connecting.**Input:**
${\hat{G}}_{c}^{\left(m,l\right)}$, $n_{\tau }$, $u_{\min}$;**Output:**
$S^{\left(m,l\right)}$;**Process:**
(a1) get $U^{F}$ of ${\hat{G}}_{c}^{\left(m,l\right)}$ by Equation (17);(a2) if $U^{F}=0$, go to (a4);(a3) find $u_{ij}^{F}=u_{\max}^{F}$;    update ${\hat{G}}_{c}^{\left(m,l\right)}$ by setting ${\hat{G}}_{c}^{\left(i,m,l\right)}={\hat{G}}^{\left(i\cup j\right)}$;    delete ${\hat{G}}_{c}^{\left(j,m,l\right)}$;    update $U^{F}$ by:        recalculating the *i*-th row of $U^{F}$;        removing both the *j*-th row and *j*-th column of $U^{F}$;    goto (a2);(a4) set $S^{\left(m,l\right)}={\hat{G}}_{c}^{\left(m,l\right)}$.

### 4.7. Missing Data Recovery

Data missing is very common in many real systems and can have a significant effect on the analysis of data. In our data acquisition project, almost all of the extracted trajectories are incomplete due to the arrival data missing randomly occurring along the stations. For the urban pubic transportation system, data missing may be caused by GPS positioning failure of the floating bus or networking problems when transmitting data. For data sampling, data missing will also happen in an unstable network. It is important that the missing data should be recovered firstly before mining the spatio-temporal properties of the trajectories.

**Figure 9:**
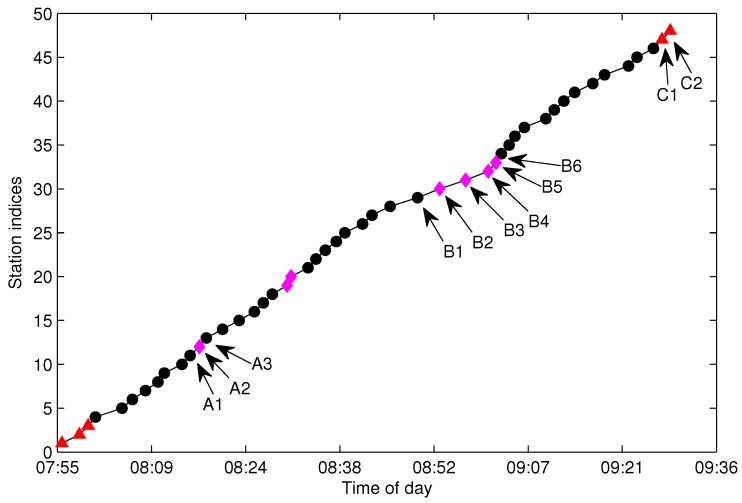
The illustration of different types of missing data.

There are tens thousands of trajectories recorded in one day for the city of Suzhou with more than 500 bus lines. Time consumed algorithms for missing data recovery are not recommended for such a system. In this work, we propose two methods of data recovery corresponding to different types of missing data, both of which are easily implemented. The first one is contextual linear interpolation for missing data recovery (CLIMDR) for the cases of missing data inside the trajectory, as shown with the magenta dots in Figure 9. The other one is median value interpolation for missing data recovery (MIMDR) for the cases of missing data outside the trajectory, as shown with the red dots in Figure 9.

The CLIMDR method is context related and statistical. Taking the points *A1-A2-A3* shown in Figure 9 as an example, the missing data of *A2* is recovered by:

(19) $$\left\{\begin{array}{c} T_{A2}=T_{A1}+t_{A1,A2}\\ t_{A1,A2}=\mathrm{CLIMDR}\left(t_{A1,A3},D_{A1,A2,A3}\right) \end{array}\right.$$

where $T_{Ai}$ is the arrival time of *Ai* for i=1,2,3, $t_{Ai,Aj}$ is the traveling time from *Ai* to *Aj* and $D_{A1,A2,A3}$ is the historical non-missing dataset selected from $S^{\left(l\right)}$ by the station indices corresponding to *A1*, *A2* and *A3*. For data operation convenience, $S^{\left(l\right)}$ (the one-route trajectories set of line *l*) can be transformed into a two-dimensional data matrix $M^{\left(l\right)}$ with $M_{i,j}^{\left(l\right)}\in M^{\left(l\right)}$ the arrival time at station *j* of the *i*-th trajectory. $D_{A1,A2,A3}$ is constructed from $M^{\left(l\right)}$ by two steps:

1. set $D_{A1,A2,A3}=M^{\left(l\right)}\left(:,\left[I\left(A1\right),I\left(A2\right),I\left(A3\right)\right]\right)$, where the first symbol “:” means selecting all rows of $M^{\left(l\right)}$, and the second parameter set “[I(A1),I(A2),I(A3)]” means selecting the corresponding columns of $M^{\left(l\right)}$ with I(Ai) the station index of Ai;
2. delete the rows of $D_{A1,A2,A3}$ when any elements in the row equal zero (missing data).

The CLIMDR method is implemented based on the assumption that $t_{A1,A2}$ and $t_{A1,A3}$ are approximately linear with:

(20) $$t_{A1,A2}=k_{1}t_{A1,A3}+k_{0}$$

In Equation (20), the parameters $k_{1}$ and $k_{0}$ are obtained with least square estimation from $D_{A1,A2,A3}$ as follows:

(21) $$\left[\begin{array}{c} k_{1}\\ k_{0} \end{array}\right]={\left({\bar{T}}_{1,3}^{T}T_{1,3}\right)}^{-1}T_{1,3}^{T}T_{1,2}$$

where $T_{1,2}$, $T_{1,3}$ and ${\bar{T}}_{1,3}$ are constructed from $D_{A1,A2,A3}$ as follows:

(22) $$\left\{\begin{array}{c} T_{1,2}=D_{A1,A2,A3}\left(:,2\right)-D_{A1,A2,A3}\left(:,1\right)\\ T_{1,3}=D_{A1,A2,A3}\left(:,3\right)-D_{A1,A2,A3}\left(:,1\right)\\ {\bar{T}}_{1,3}=\left[T_{1,3}\;\;1\right] \end{array}\right.$$

The CLIMDR method for the case of *A1-A2-A3* shown in Figure 9 is called CLIMDR-1, which means recovering one depth missing datum, i.e., the number of successive missing data is one. Similarly, we call CLIMDR-*n* for the *n*-depth missing data recovery by the CLIMDR method. CLIMDR-*n* is implemented by an iterative method. Taking the trajectory points *B1-B6* shown in Figure 9 as an example, there are four missing data in *B1-B6*; hence, the missing depth is four, and the corresponding recovery method is CLIMDR-4. In the interpolating procedure, we first recover *B2* by $T_{B1}$ and $D_{B1,B2,B6}$; then, *B3* can be recovered by $T_{B2}$ and $D_{B2,B3,B6}$. The interpolation procedure continues until *B5* is recovered.

The MIMDR method is applied for the cases of missing data outside the extracted trajectory. In those cases, most of interpolation methods, including linear interpolation, kinematic interpolation [23], Bezier curves [24], Catmull-Rom curves [25] and our proposed CLIMDR, are not available, since the head part or tail part of the data is also lost. In the MIMDR method, we firstly partition the data into the weekday set and weekend set. Then, we split the time of day into time slots with equal duration of 20 min and calculate the median value of traveling time in every time slot from the historical non-missing dataset. Finally, the missing data are recovered by the median value according to the time slot and weekday/weekend attributes. In practice, we only recover the starting and terminal stations of the trajectory by MIMDR to avoid accumulative errors. When the missing head or tail of the trajectory is recovered, the CLIMDR method can be applied again to recover the middle part of the trajectory.

## 5. Results and Discussion

### 5.1. Comparing Trajectory Clustering between F-FCM and B-FCM

**Figure 10:**
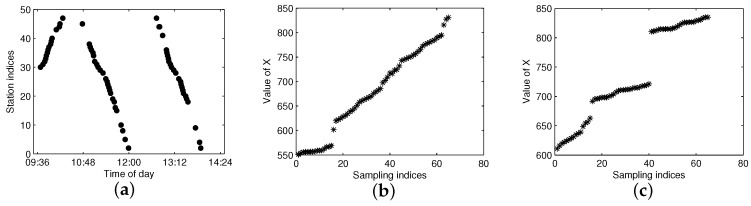
Comparison of the separable performance of *X* constructed by forward and backward clustering of $G^{\left(m,l\right)}$ with wrong traveling direction trajectories. (**a**) Arrival events; (**b**) constructed *X* for forward clustering; (**c**) constructed *X* for backward clustering.

The F-FCM method is suitable for the trajectories that have the right traveling direction, whereas the B-FCM method is suitable for those that have the wrong traveling direction. In practice, it is difficult to determine whether the bus arrival dataset $G^{\left(m,l\right)}$ contains wrong direction trajectories. An alternate solution is to partition $G^{\left(m,l\right)}$ by both F-FCM and B-FCM and then select the better partition according to the objective function in Equation (1). Figure 10 shows the comparison of the separability for the constructed forward feature and backward feature of $G^{\left(m,l\right)}$, which contains wrong traveling direction trajectories. As shown in Figure 10b,c, when $G^{\left(m,l\right)}$ contains wrong direction trajectories, the separable performance of *X* constructed by the forward feature is worse than that by the backward feature.

**Figure 11:**
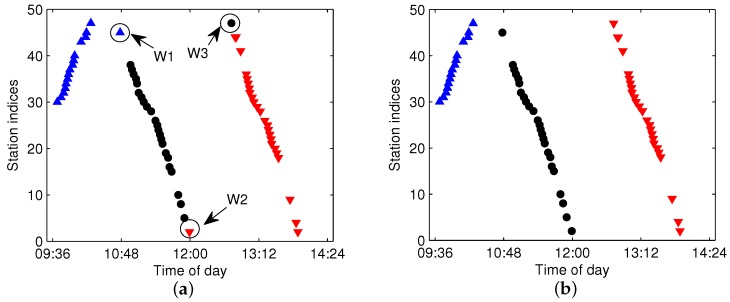
The comparison of the aggregated clusters by both forward FCM (F-FCM) and backward FCM (B-FCM). (**a**) Clustering by F-FCM; (**b**) clustering by B-FCM.

The dataset $G^{\left(m,l\right)}$, shown in Figure 10, is used as an example. Both F-FCM and B-FCM with a=2 and c=3 are applied, and the aggregated clusters are shown in Figure 11. For F-FCM, the constructed *X* does not have much of a partitioning margin between the different clusters, thus resulting in wrong partitions for the data *W1*, *W2* and *W3*, which are pointed out in Figure 11a. In contrast, the B-FCM separates the three clusters correctly. The objective functions calculated by Equation (1) for the above approaches are $J_{a}^{F-FCM}=3.2\times 10^{4}$ and $J_{a}^{B-FCM}=6.2\times 10^{3}$. Since $J_{a}^{B-FCM}<J_{a}^{F-FCM}$, the clustering results by B-FCM should be chosen rather than F-FCM. We consider the clustering approach selection criterion ruled by $J_{a}$ to be more natural and feasible than the straight way of directly checking whether there are wrong direction trajectories in the dataset.

### 5.2. Discussing the Setting of Cluster Number for FCM

**Figure 12:**
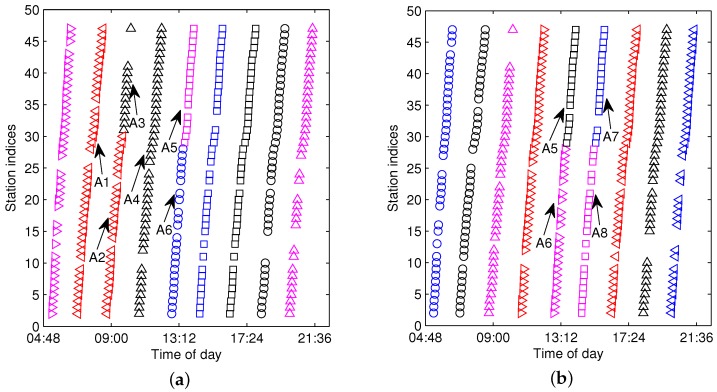
The comparison of FCM with different settings of *c*. (**a**) a=2, c=9; (**b**) a=2, c=11.

The parameter *c*, which is also called the cluster number, is an important setting for the FCM method. Figure 12 shows the comparison of FCM clustering with different settings of *c*. In the figure, the scanned $c_{0}$ by Equation (9) is the same as the true count of the trajectories, i.e., $c_{0}=c_{0}^{*}=9$. By setting $c=c_{0}$, the F-FCM clustering in Figure 12a reveals that *A1–A2* and *A3–A4* are incorrectly partitioned. To fix this wrong clustering, *A2* should be split out from the *A1–A2* cluster, and *A3* should be split out from the *A3–A4* cluster. Then, *A3* should be connected to *A2*. In comparison, when *c* is set as c=11 with α=1.3 in Equation (10), Figure 12b reveals that the wrong partitions for *A1–A2* and *A3–A4* in Figure 12a are rectified. The drawback of enlarging the gain parameter *α* is that one trajectory may be split into several different clusters, e.g., *A5–A6* and *A7–A8* in Figure 12b. When *c* is small, every cluster must be checked, and the over-aggregated cluster must be split into two or more trajectories. Then, some of the trajectories must be connected into one. In contrast, when *c* is large, it is only necessary to perform the connecting work for some small clusters. We prefer to set *c* as greater than the actual count of trajectories in order to avoid the problem of over-clustering.

The proposed FCM method solves the problem of deciding which data should be classified into a cluster as a trajectory; however, it cannot distinguish which data in the cluster are abnormal and should be eliminated. Thus, anomaly detection and erroneous data elimination are necessary for the aggregated clusters.

### 5.3. Testing of the Trajectory Fragment Cleaning Algorithm

**Figure 13:**
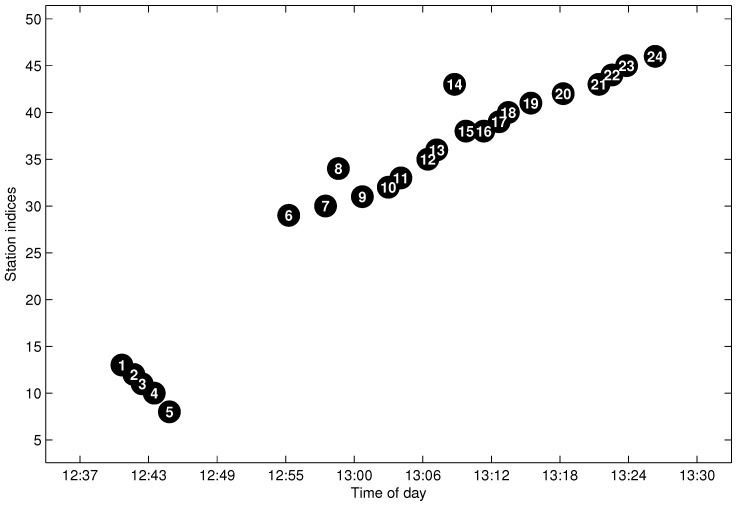
The aggregated trajectory fragment with noisy data.

To illustrate the cleaning algorithm, we give a real trajectory that was sampled on 29 September 2012 for the line l=130, as shown in Figure 13. The sampling date is the last working day before a week-long holiday in China; thus, it is also a traffic jam day. The bus company scheduled more buses for some busy lines on that day in order to satisfy the travel demand. However, the position reporting devices may not work well for some temporarily-scheduled buses, which results in a greater number of outliers on that day. Table 2 gives the detailed arrival data, which are shown in Figure 13. In the figure, the arrival data labeled as $P_{2}$–$P_{5}$, $P_{8}$ and $P_{14}$ are easily distinguishable as outliers by our empirical knowledge. Furthermore, $P_{16}$ is reasoned to be a candidate outlier when compared to $P_{15}$. By applying the proposed AD method, all of the erroneous data are detected and eliminated from the trajectory.

**Figure 14:**
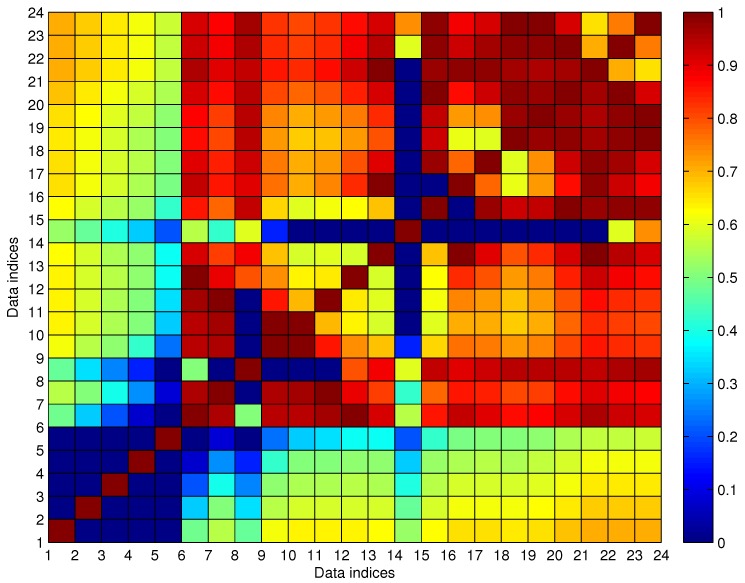
The evaluated fuzzy connecting membership function (MF) matrix for the trajectory fragment shown in Figure 13.

To better illustrate the process of Algorithm 3, the data shown in Figure 13 are used as an example. Figure 14 shows the colorized $U^{c}$ for the data given in Table 2, with the parameters given in Equation (15). As shown in the figure, the abnormal data $P_{2}$–$P_{5}$, $P_{8}$ and $P_{14}$ show a lower connecting MF than others.

**Figure 15:**
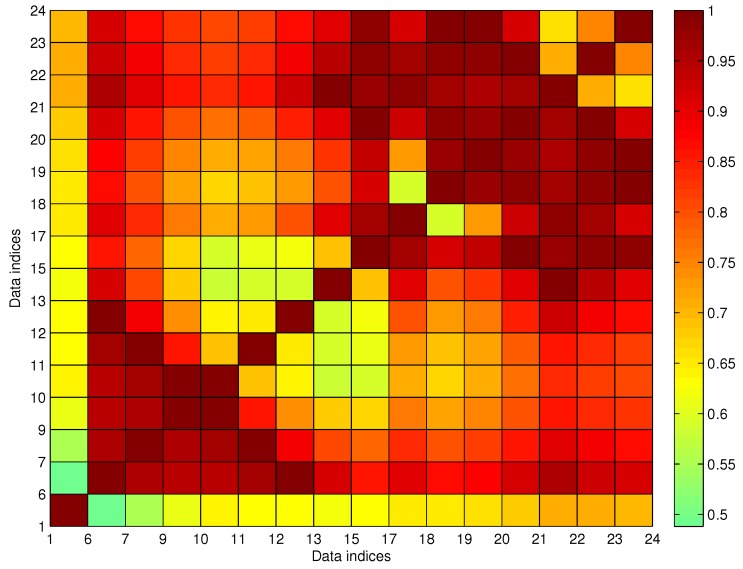
The fuzzy connecting MF matrix for the trajectory fragment after outlier elimination.

**Figure 16:**
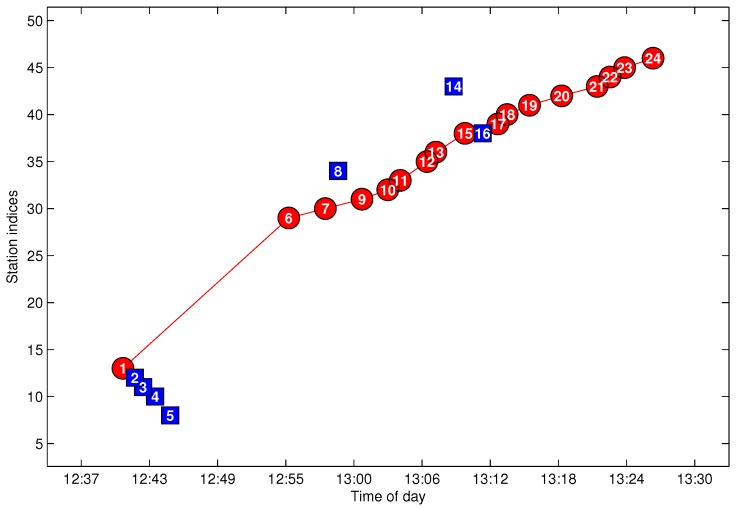
The cleaned trajectory fragment.

In the first round, k* = 14 with ${\bar{N}}_{14}^{c}=13$; thus, $P_{14}$ is chosen as the noisy data and is deleted in this round. In Algorithm 3, one noisy datum is selected for deletion each round until ${\bar{N}}^{c}=0$. In Figure 14, both $P_{15}$ and $P_{16}$ satisfy the connecting possibility threshold to other non-noisy data; however, $P_{15}$ and $P_{16}$ are in the same station index, which means that at least one of them should be classified as noisy data. We prefer to delete the data with the lower average connecting possibility, which results in maximizing $\mathrm{avg}({\hat{U}}^{c})$. In the eighth round, both $P_{15}$ and $P_{16}$ have the same anomalous indexing, and after comparing the row summation of $U^{c}$, $P_{16}$ is discriminated as the noisy data. Finally, in the ninth round, ${\bar{N}}^{c}=0$ is obtained, which means the noise detection process is complete. The outputted ${\hat{U}}^{c}$ is illustrated in Figure 15. As shown in the figure, all of the data are with $u\left(t\right)>u_{\min}$ to each other, and the corresponding ${\hat{G}}_{c}^{\left(j,m,l\right)}$ is a cleaned trajectory fragment. Additionally, if any one of the deleted data is added to ${\hat{G}}_{c}^{\left(j,m,l\right)}$, the corresponding ${\bar{N}}^{c}\neq 0$. Thus, the size of ${\hat{G}}_{c}^{\left(j,m,l\right)}$ is maximized by Algorithm 3. Figure 16 shows the final results according to the data given in Figure 13, where the red labels indicate the cleaned data ${\hat{G}}_{c}^{\left(j,m,l\right)}$ and the blue ones indicate noisy data ${\hat{R}}_{c}^{\left(j,m,l\right)}$.

### 5.4. Testing of the Trajectory Fragments Connecting Algorithm

**Figure 17:**
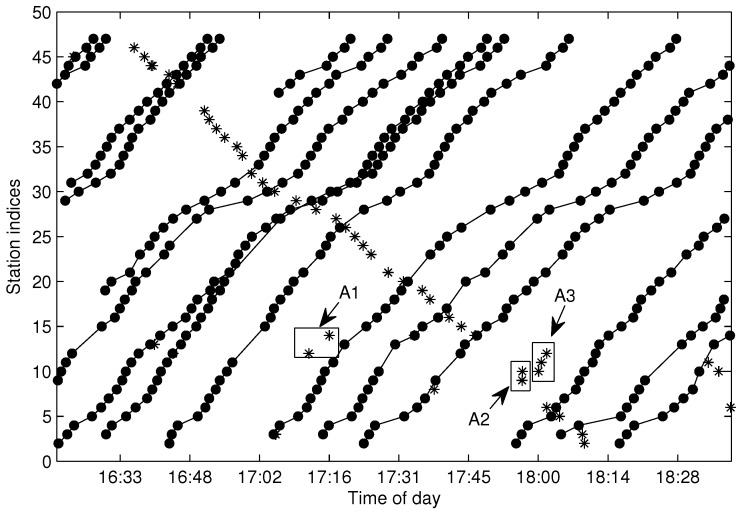
The cleaned trajectories after trajectory fragments connecting (TFC).

**Figure 18:**
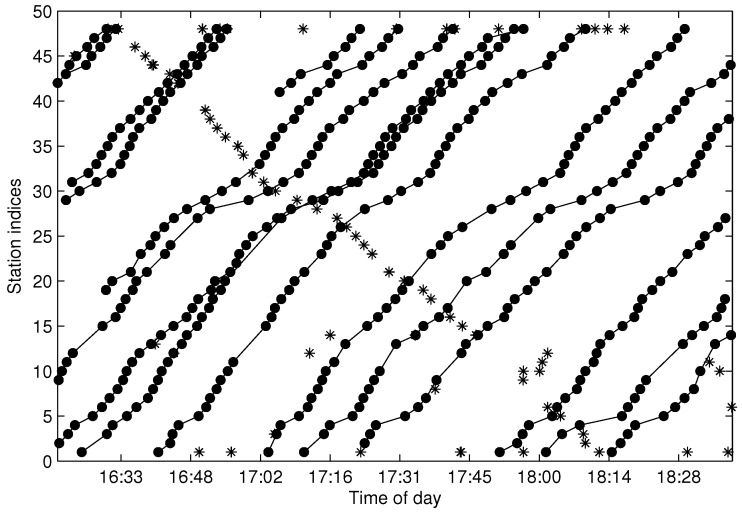
The final extracted trajectories after adding the arrival data of the first and final stations (FF).

When the traveling data are aggregated by FCM and cleaned by the proposed cleaning algorithm, the following procedure is to connect the over split clusters into a one-route trajectory, which is implemented by the trajectory fragments connecting (TFC) algorithm. The dataset shown in Figure 6 is utilized for testing. Figure 17 shows the cleaned and connected trajectories after TFC, where the data labeled with “*” are the noisy data, and the connected dot data are one-route trajectories (also denoted by $S^{\left(m,l\right)}$). In the figure, the data in the first and final stations (FF-data) are omitted. Furthermore, the trajectories labeled by *A1*, *A2* and *A3* are filtered as noisy data because their respective sizes violate the connecting rule described by Constraint (18). When all of the one-route trajectories are extracted by Algorithm 4, it is easy to add the available FF-data to $S^{\left(m,l\right)}$ by u(t) between the FF-data and the data in $S^{\left(m,l\right)}$. Figure 18 shows the final extracted trajectories from the data shown in Figure 4. As illustrated in the figure, all of the one-route trajectories are extracted and cleaned.

### 5.5. Performance of the Missing Data Recovery Methods

A dataset with a size of 8288 one-route trajectories for the line l=130 was used to verify the performance of our proposed missing data recovery methods. The dataset was sampled from 1 August–10 December in 2012 and was extracted by the addressed extracting algorithm. There are 118,917 (29.89%) data lost in the trajectories. For a real system, it is difficult to conduct the studies such as dynamic headway mining [31,32], service reliability evaluation [33], scheduling analysis [34,35,36], and so on, before missing data are interpolated, especially for the trajectories in the high missing rate. Thus, missing data recovery is important for the trajectory mining of urban buses. The proposed CLIMDR method is easily implemented and outperforms when the dataset is large enough (thousands of trajectories or more).

**Figure 19:**
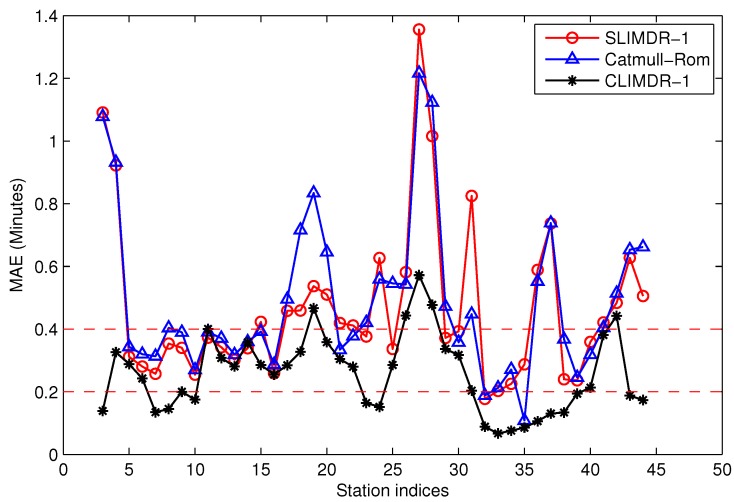
Comparisons of the MAE performances of the recovered data by contextual linear interpolation for missing data recovery (CLIMDR)-1.

Figure 19 shows the mean absolute error (MAE) of the recovered data by CLIMDR-1 from Station 3–Station 46 for the tested dataset. For comparison, the traditional straight linear interpolation for missing data recovery with one depth missing data (SLIMDR-1) and Catmull–Rom cubic interpolation [25] are also conducted. As compared in the figure, the performance of our method is better than those of the other two methods for almost allstations, especially for the stations with congested roads and a large MAE.

**Figure 20:**
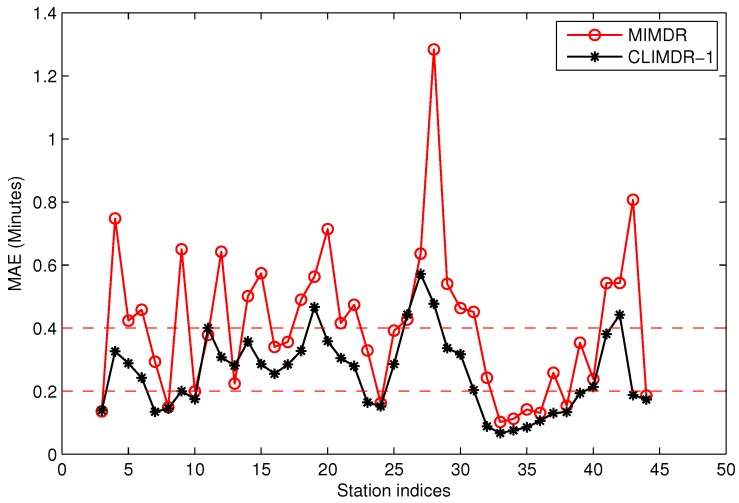
The MAE performance of the recovered data by median value interpolation for missing data recovery (MIMDR).

Figure 20 shows the MAE performance of the recovered data by MIMDR for the same dataset. In Figure 20, there are a total of 42 stations (from Station 3–Station 44) for the test. For the MIMDR method, there are 10 stations with MAE≤0.2 and 21 stations with MAE≤0.4. In contrast, the CLIMDR-1 method outperforms MIMDR by 17 stations with MAE≤0.2 and 36 stations with MAE≤0.4. For the real system, we only recover the first and final stations for the trajectories by the MIMDR method, and then, we apply the CLIMDR method to interpolate the middle parts for the trajectories.

**Figure 21:**
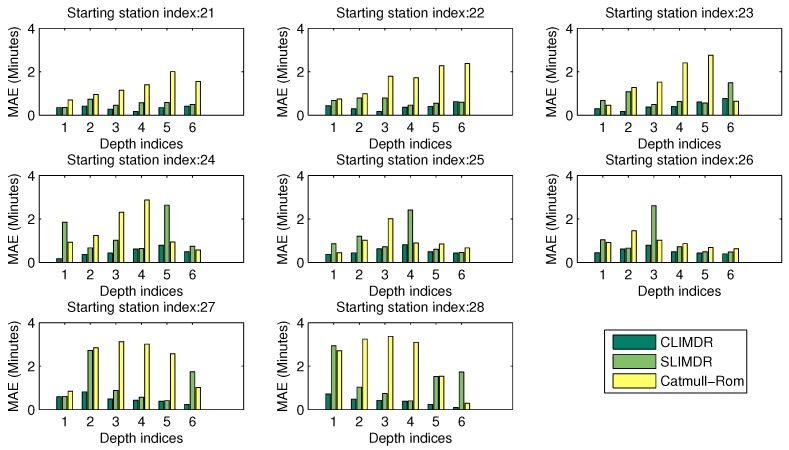
Comparisons of the MAE performances for six-depth missing data recovery.

Missing consecutive data also commonly occur in the real system. The CLIMDR-*n* method is suitable for the cases where *n* consecutive data are missing in the trajectories, and it outperforms comparable methods in most cases. Figure 21 shows the comparisons of the MAE performances for the cases of six consecutive missing data, i.e., six-depth missing data. We selected eight cases, for which the starting station is from 21–28, to test the performance of CLIMDR-6. In the figure, the MAE performances of the three methods are grouped, where the left bar represents the proposed CLIMDR method, the middle bar represents the SLIMDR method and the right bar represents the Catmull–Rom method. For six-depth missing data recovery, there are six groups of comparisons in total for each case. As shown in the figure, group *i* (i=1,2,⋯,6) also indicated the depth index, representing the performance of the corresponding *i*-th missing data in the six-depth missing data. As illustrated in the figure, the CLIMDR method outperformed the other two methods in almost all of the cases.

**Figure 22:**
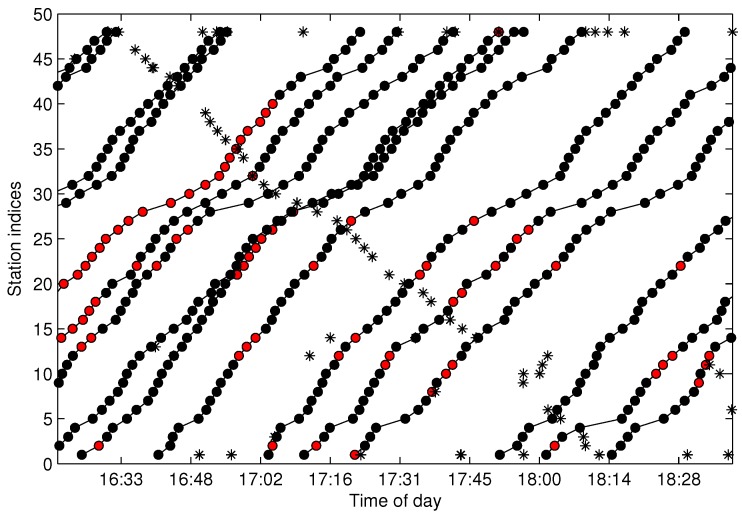
The final recovered trajectories.

Figure 22 shows the final recovered trajectories for the same dataset as illustrated in Figure 17, where the red dots represent the recovered data. When the cleaned bus trajectories are extracted and recovered for the missing data, the data are stored in the form of one trajectory for one record, and all of the data from different sampling days are merged into one database. In the final database, the trajectories are sorted by dispatching time and can be retrieved in the form of a two-dimensional matrix, where one row represents one trajectory. One merit of the trajectory matrix is that the traveling time set between any two stations can be easily derived by subtracting the corresponding two columns from the matrix. Furthermore, the headway dynamics can also be calculated by subtracting any two adjacent row data in the matrix.

## 6. Conclusions

In this paper, we have presented a novel framework for urban bus traveling data acquisition and data processing for data sampled from the Internet. The work consisted of two parts: trajectory extraction with noisy data removal and missing data recovery. We introduced the pentagonal fuzzy membership function for the purpose of describing the connecting possibility of arrival events. The fuzzy membership function was extended to be a matrix form and was applied to detect the outliers in a cluster. Furthermore, the connecting rule for clusters was designed with the help of fuzzy MF. The trajectory extraction work was implemented by three sub-procedures: data clustering by FCM, trajectory fragments cleaning and trajectory fragments connecting.

In real systems, the extracted trajectories have a high rate of missing data for data sampled from the Internet. We classified the missing data into two types: missing inside the trajectory and missing outside the trajectory. We introduced the CLIMDR method for data interpolation for data missing inside the trajectory and the MIMDR method for recovering the head or tail of the trajectory. We showed that the proposed CLIMDR method outperformed the straight linear interpolation method and Catmull–Rom interpolation method in most cases. The CLIMDR method is data driven and parameter free, which is easily implemented and robust.

In the future, we plan to investigate headway dynamics, scheduling efficiency and traveling time prediction of urban buses with the data sampled from the Internet and preprocessed by the methods given in this paper.

## Figures and Tables

**Table 1:** Illustration of arrival data stored in the “ArrivalData” table.

ID	LineCode	StationIndex	BusIndex	ArrivalTime
1	158	3	1	584.2000
2	158	7	2	583.0167
3	158	12	3	584.8333

**Table 2:** The arrival data shown in Figure 13.

Label	Station Index	Arrival Time	Label	Station Index	Arrival Time
P1	13	761.0	P13	36	788.0
P2	12	762.1	P14	43	789.5
P3	11	762.8	P15	38	790.5
P4	10	763.8	P16	38	792.0
P5	8	765.1	P17	39	793.3
P6	29	775.3	P18	40	794.1
P7	30	778.5	P19	41	796.0
P8	34	779.6	P20	42	798.8
P9	31	781.6	P21	43	801.8
P10	32	783.8	P22	44	803.0
P11	33	784.9	P23	45	804.2
P12	35	787.2	P24	46	806.7

## Supplementary Materials

The following are available online at http://www.mdpi.com/1424-8220/17/2/342/s1.

## Appendix A

The raw bus arrival data formed like Table 1 for l=130 are available as a supplementary file, which sampled during 1 August to 9 December 2012. The extracted and interpolated trajectories formed as $S^{\left(l\right)}$ for l=130 are also available. All of the data are stored in the form of one file for one sampling day.
